# Process signatures in glatiramer acetate synthesis: structural and functional relationships

**DOI:** 10.1038/s41598-017-12416-1

**Published:** 2017-09-21

**Authors:** Víctor R. Campos-García, Daniel Herrera-Fernández, Carlos E. Espinosa-de la Garza, German González, Luis Vallejo-Castillo, Sandra Avila, Leslie Muñoz-García, Emilio Medina-Rivero, Néstor O. Pérez, Isabel Gracia-Mora, Sonia Mayra Pérez-Tapia, Rodolfo Salazar-Ceballos, Lenin Pavón, Luis F. Flores-Ortiz

**Affiliations:** 1 Unidad de Investigación y Desarrollo, Probiomed S.A. de C.V., Cruce de Carreteras Acatzingo-Zumpahuacán s/n, Colonia Los Shiperes, Tenancingo, 52400 Estado de México, Mexico; 2 Unidad de Desarrollo e Investigación en Bioprocesos (UDIBI), Escuela Nacional de Ciencias Biológicas, Instituto Politécnico Nacional, Prolongación de Carpio y Plan de Ayala s/n, Colonia Santo Tomás, 11340 Ciudad de México, Mexico; 3 Departamento de Farmacología, Cinvestav-IPN, Avenida Instituto Politécnico Nacional 2508, Colonia San Pedro Zacatenco, 07360 Ciudad de México, Mexico; 4 Departamento de Química Inorgánica y Nuclear, Facultad de Química, Universidad Nacional Autónoma de México (UNAM), Ciudad Universitaria, Investigación Científica 70, 04510 Ciudad de México, Mexico; 5 Unidad de Investigación, Desarrollo e Innovación Médica y Biotecnológica (UDIMEB), Escuela Nacional de Ciencias Biológicas, Instituto Politécnico Nacional, Prolongación de Carpio y Plan de Ayala s/n, Colonia Santo Tomás, 11340 Ciudad de México, Mexico; 6 Laboratorio de Psicoinmunología, Dirección de Investigaciones en Neurociencias, Instituto Nacional de Psiquiatría Ramón de la Fuente, Calzada México-Xochimilco 101, Colonia San Lorenzo Huipulco, 14370 Ciudad de México, Mexico

## Abstract

Glatiramer Acetate (GA) is an immunomodulatory medicine approved for the treatment of multiple sclerosis, whose mechanisms of action are yet to be fully elucidated. GA is comprised of a complex mixture of polypeptides with different amino acid sequences and structures. The lack of sensible information about physicochemical characteristics of GA has contributed to its comprehensiveness complexity. Consequently, an unambiguous determination of distinctive attributes that define GA is of highest relevance towards dissecting its identity. Herein we conducted a study of characteristic GA heterogeneities throughout its manufacturing process (process signatures), revealing a strong impact of critical process parameters (CPPs) on the reactivity of amino acid precursors; reaction initiation and polymerization velocities; and peptide solubility, susceptibility to hydrolysis, and size-exclusion properties. Further, distinctive GA heterogeneities were correlated to defined immunological and toxicological profiles, revealing that GA possesses a unique repertoire of active constituents (epitopes) responsible of its immunological responses, whose modification lead to altered profiles. This novel approach established CPPs influence on intact GA peptide mixture, whose physicochemical identity cannot longer rely on reduced properties (based on complete or partial GA degradation), providing advanced knowledge on GA structural and functional relationships to ensure a consistent manufacturing of safe and effective products.

## Introduction

Non-Biological Complex Drugs (NBCDs) are defined as medicinal products not derived from living sources whose active substance is not a homo-molecular structure but rather a mixture of closely related structures that cannot be fully isolated, quantitated, characterized or described by physicochemical analytical means^1^. Despite being chemically synthesized, NBCDs resemble biologics more closely than small-molecule drugs because of their larger molecular masses and higher order structures, being equally or more complex than most biopharmaceuticals. Examples of NBCDs include iron-carbohydrate complexes, glatiramoids, liposomes, polymeric micelles, swelling polymers and so-called nanomedicines^2^.

Given the inner complexity of NBCDs, the control and consistency of their manufacturing process is particularly relevant, as it defines their final composition, quality and *in vivo* performance^3^. A major archetypal NBCD is Glatiramer Acetate (GA), an immunomodulatory medicine approved for the treatment of multiple sclerosis (MS), which slows the progression of disability and reduce the frequency of relapses in ambulatory patients with relapsing remitting multiple sclerosis and in patients with high risk to develop clinically definitive multiple sclerosis^4^.

Indeed, it is not surprising to affirm that GA has an intricate physicochemical and biological nature; able to activate the immune systems of many individuals and effectively treat the highly heterogeneous disease that is MS. This medicine is comprised of a complex mixture of linear and random polypeptides of L-glutamic acid, L-lysine, L-alanine and L-tyrosine with an average molar ratio of 0.141, 0.338, 0.427 and 0.095, respectively^5^.

It is worth to notice that GA biological effect on MS, assessed on an experimental autoimmune encephalomyelitis (EAE) model, was unforeseen during early tests conducted by Prof. M. Sela and coworkers^6^. GA was first thought to be an EAE inducer rather than a suppressor^7^ as it was synthesized with an analogous amino acid composition to myelin basic protein, an encephalitogenic agent^8^. Further studies in a variety of animals, including rabbits^9^, mice^10^, rhesus monkeys^11^, baboons^12^ and, ultimately, humans^13^, showed that the suppressive effect of GA was not restricted to a particular species.

Moreover, the precise molecular mechanisms of action of GA that contribute to its pharmacological activity are yet to be fully elucidated, although some insights on the immunomodulatory effects of GA at distinct levels of the immune cascade have been reported^14^. For instance, some studies revealed the presence of anti-GA-specific antibodies in the serum of treated patients and animals^15–18^. However, these antibodies have an unclear effect, it has been suggested that they do not alter or even promote the clinical effect of GA^15,19^.

Besides its multiple modes and effects over the immune system, GA biological outcomes are well described and have been well established from its complete polypeptide mixture. In this sense, an atypical physicochemical profile of GA such as an altered aggregation behavior, secondary and tertiary structural changes or differences in its molecular mass or charge heterogeneities may lead to a different immunological or toxicological responses. For example, an analogous product of GA with the same amino acid composition but with a higher molecular mass distribution (TV-5010, Protiramer) induced severe injection site reactions accompanied by kidney and liver damage in rats and monkeys during long term non-clinical toxicity studies, which led to termination of the drug development program^20,21^.

Given the current knowledge on GA, certainly, a well defined and consistent manufacturing process is a key element to ensure the maintenance of the drug’s safety, efficacy and tolerability, while reducing the potential incidence of augmented immunological risks^22,23^, or an enhanced toxicity profile^20,21^. Hence, a thorough understanding of process critical parameters and their control, which ultimately determine the specific GA physicochemical properties and biological effects, would be of highest relevance towards dissecting the complex nature of this medicine.

Although, a fundamental reaction protocol for the synthesis of GA is publicly available^5,7,24–34^, information regarding the relationships among each manufacturing condition and the physicochemical properties of the synthesized peptides is rather scarce or is limited by the intellectual property right of trade secret, contributing to the GA comprehensiveness complexity as a pharmaceutical product.

GA complexity may impede the complete identification of the physicochemical and biological properties utterly responsible of its efficacy and safety^35–43^, but it does not preclude a finding of distinctive attributes that define GA towards dissecting its identity and improve the understanding of the biological and clinical properties of glatiramer acetate^44^. For instance, GA highly disperse molecular mass distribution has been characterized, ranging from ~3 kDa to ~45 kDa with a mass-average molar mass mean (M_w_) of ~10.5 kDa^45^, along its highly disperse electric charge distribution that ranges from ~1 to more than 60(e) with a mean net positive charge of ~48(e)^46^.

The need of scientific knowledge around this medicine demands a documented study of the impact on GA molecular properties of the process conditions during each manufacturing stage, including: (1) Polymerization of N-carboxy-α-amino acid anhydrides (NCAs) in anhydrous dioxane, with diethylamine (DEA) acting as the polymerization initiator, (2) Depolymerization in glacial acetic acid using hydrogen bromide along with a deprotection step in aqueous piperidine, followed by (3) Dialysis and lyophilization steps to produce the final acetate salt of the copolymer, and (4) Formulation of the pharmaceutical ingredient with a mannitol solution^5,7,24–34^.

The relationships between manufacturing conditions and physicochemical properties of GA can be deciphered experimentally since they lie on reaction kinetics that govern the manufacturing process; for instance, changes during the polymerization stage would lead to altered amino acid compositions along each growing peptide^47^, as reactivities of NCAs, initiation rates and elongation rates may differ. Besides, changes in the final dialysis membrane will undeniably produce differences in the known molecular mass Poissonian-like distribution of GA^48^.

However, an important technical problem is to analyze GA by methods that could unambiguously reveal its attributes without perturbing its populations. To date, preferred methods include the estimation of reduced properties, such as global amino acid molar ratios, mean molar mass or local amino acid abundances (N-terminal and C-terminal)^5,7,24–34,49,50^. Nevertheless, these methods solely cannot evaluate GA complexity, neither support the establishment of its physicochemical identity as a whole, as mere amino acid relative proportions and mean molecular mass determinations do not give enough information to fully trace GA peptide distribution and advance in our understanding of GA manufacturing process. Thus, it is essential to assess GA complexity by more capable methods and to study the impact of each process variable towards defining its chemical attributes, hereafter called GA process or structural signatures^5^.

Herein, we study the critical process parameters of GA through a set of tailored copolymer batches produced to systematically investigate how the structural signatures of GA were affected; by using known analytical principles on specifically designed methodologies to meet this purpose. Moreover, an *in vivo* study was performed to assess the immunological effects of the distinctive GA heterogeneity and altered profiles, while a toxicology profile (acute and with multiple doses) was evaluated to confirm its safety profile. Our results provide comprehensive evidence of the impact of critical GA manufacturing process variables towards dissecting GA complexity. Ultimately these findings are intended to fulfill the scarcity of crucial knowledge for this medicine, which could lead to the development of novel or generic polypeptide MS treatments.

## Results and Discussion

As noted, GA complexity comprises undefined chemical species (i.e., polypeptides with different amino acid sequences) and multiple structures, which are neither possible nor practical to be independently analyzed before use and upon administration. Besides, GA intrinsic heterogeneity is utterly responsible for its immunological, pharmacological and toxicological profiles; thus confirming the relationship between its identity and biological activity. In this regard, is mandatory to use suitable techniques to differentiate among closely related profiles against the distinctive heterogeneity profile of GA, without missing its minor populations^46^. We developed SE-UPLC^45^, SCX-UPLC and RP-UPLC methodologies^46^ to evaluate molecular mass, electric charge and hydrophobicity distributions as the most reliable characteristics of the whole mixture of amino acid sequences and peptide lengths of GA through each stage of the manufacturing process (viz., polymerization, depolymerization, deprotection and purification) in order to assess critical process parameters and their impact on GA structural signatures.

All experiments were modified from the standard synthesis conditions (STD) used for GA manufacturing. GA produced by STD (GA-STD, Probioglat) has been extensively characterized in terms of its distinctive and equivalent molecular mass^45^; electric charge and hydrophobicity^46^; and refractive index increment and extinction coefficient^51^ with respect to the reference medicinal product (Copaxone).

### Initiator concentration is related to the abundance of GA larger and more basic peptides

GA composition is defined largely by the conditions used during the polymerization stage, which is initiated by individual reactions of single NCAs with DEA. These reactions generate polymerization cores that are further elongated into polypeptide chains through NCAs incorporation. In this sense, NCAs initial ratio would determine the final amino acid molar proportions of GA, but not its specific sequences and structures. In effect, amino acid sequences and structures of the synthesized polypeptides are result of polymerization conditions, such as temperature and DEA proportion. However, to our knowledge, structural signatures of GA cannot be foreseen beyond NCAs consumption kinetics at different polymerization conditions, final amino acids proportions or few sequencing cycles at the C- and N-peptide termini. In order to address this issue, resultant charge and mass heterogeneities of the peptide mixtures coming from modified polymerization conditions were evaluated, bringing traceable and global endpoints to the obtained mixtures of sequences at different conditions.

**Figure 1:**
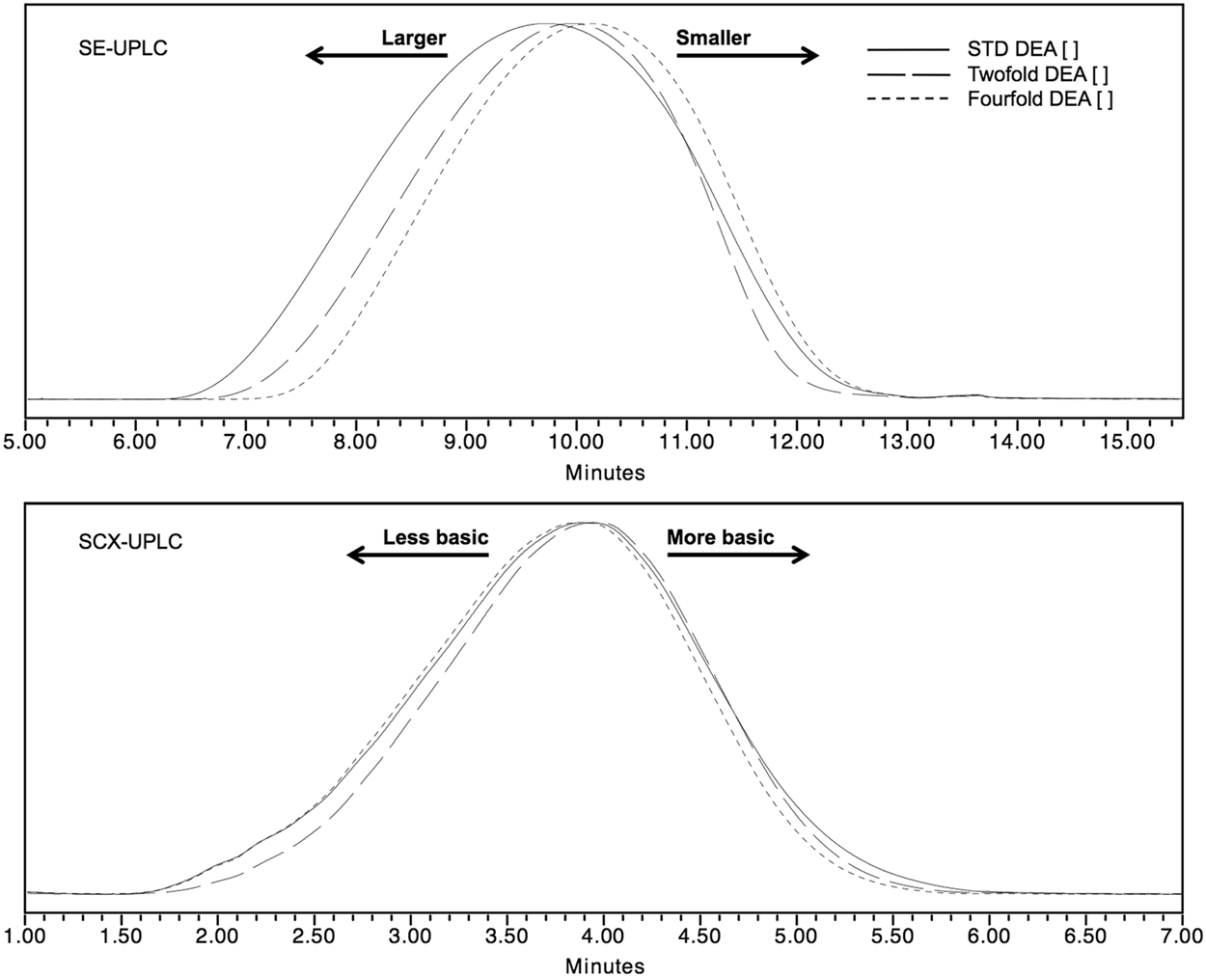
STD, twofold and fourfold DEA experiments analyzed by SE-UPLC and SCX-UPLC.

Based on STD, experiments using twofold and fourfold initiator (DEA) concentrations were performed while maintaining the rest of the manufacturing parameters unchanged. These modifications altered the reaction kinetics since initiator availability was increased for both experimental conditions, as seen by differences on the obtained heterogeneity profiles. Results showed that DEA concentration is inversely related to the abundance of large and more basic peptides (Fig. 1). Besides, a dispersity reduction (i.e., narrowed distribution) of size and charge heterogeneity profiles was observed as DEA concentration increased. It is important to notice that a positive correlation between molecular mass and electric charge was a constant observation for most GA process-modified batches, as expected due to the composition of GA: the higher the molecular mass of the peptides, the higher probability of lysine incorporation and the higher the positive electric charge will be. This agreed well with previous results reported by our group^46^.

The impact of DEA on the abundance of larger and more basic peptides is related to a higher formation of synthesis cores as the availability of DEA is increased. Otherwise, this could be explained in terms of degree of NCAs polymerization (DP)^52–54^, where DP is inversely proportional to the number of synthesized polymer chains at a fixed monomer (i.e., NCAs) concentration. Further, DP is related to the ratio of polymer chains elongation and synthesis core formation velocities. In effect, at a fixed monomer concentration, the higher number of synthesis cores the higher competition against monomers, resulting in a higher number of polymers with a lower molecular mass and *vice versa*.

This behavior has been observed for the synthesis of polyL-lysine, poly(γ-benzyl-L-glutamate) (PBLG) and poly(γ-ethyl-L-glutamate) homopolymers using different initiators including DEA^55–61^.

### NCAs concentration is related to abundance of GA smaller and less basic peptides

As stated, once polymerization begins by reactions of single NCAs with DEA, it is followed by the reaction of the N-terminus of single NCAs with each growing polymer chains. The propagation process is governed by the relative amount of each NCA and its differential reaction kinetics against the polymer chains. This synthesis behavior, known as propagational shift^62^, emphasizes the use of a specific NCAs molar ratio, order of addition and molar concentration towards obtaining the desired characteristics in GA.

A previous work performed by our research group showed the impact of the NCAs order of addition on GA physicochemical properties, even though the global NCAs molar ratio was maintained, altered heterogeneity profiles of molecular mass, charge, hydrophobicity and ultimately the loss of GA identity^46^ were observed. In effect, by altering the order of addition of each NCA during polymerization, the known variation of the amino acid molar fractions along the growing copolymer was modified and results in altered heterogeneity distributions of molecular mass and electric charge. This highlights the importance of NCAs availability during the polymerization process.

**Figure 2:**
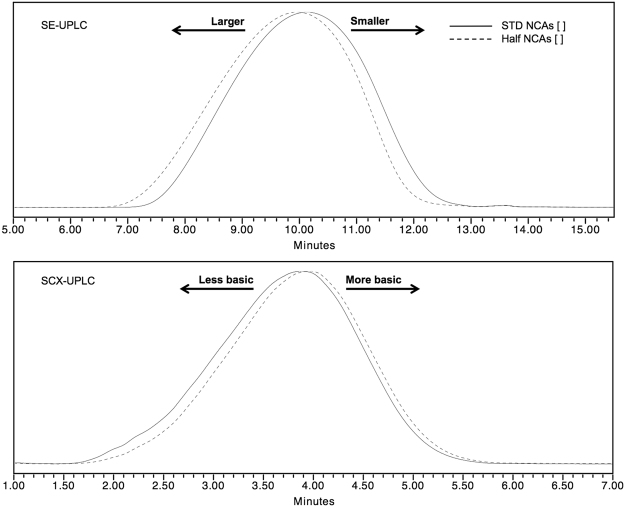
STD and half NCAs concentration experiments analyzed by SE-UPLC and SCX-UPLC.

On this regard, by modifying NCAs molar concentration a correlation towards the abundance of smaller and less basic peptides was found. Chromatographic profiles of a copolymer batch synthesized from a NCAs mixture at 1:2 dilution (half NCAs concentration) while keeping the other manufacturing parameters unchanged (including a fixed NCAs order of addition, molar ratio and DEA proportion) showed a shift in retention time to larger and more basic populations in comparison to the STD (Fig. 2). Moreover, no evident changes were detected between the polydispersity of STD and the half NCAs concentration batch.

It is thought that the occurrence of larger and more basic peptides at lower NCAs concentration is associated to a lower collision probability against DEA, as less polymer cores are more elongated with respect to STD at total NCAs conversion. Although NCAs and DEA concentrations were diminished, DEA proportion is almost 50 times less in comparison to NCAs, thus favoring elongation kinetics instead of core establishment velocity.

Similar results have been reported for PBLG using triethyamine as initiator, since the homopolymers molecular weight was inversely related to the monomer concentration^60^; or using dicyclohexylamine as initiator, where a lower collision probability was suggested to explain the maintenance of homopolymers molecular weight at lower monomer concentrations, clearly at the expense of lower NCAs conversion percentages^61^.

### Polymerization temperature is related to GA distribution broadening

Once NCAs and DEA are mixed, polymerization occurs under room temperature (RT) for at least 24 h to achieve total NCAs conversion. However, it is expected that NCAs polymerization begins at initial mixing and determines the number of resulting polymer cores and their properties, depending on the reaction kinetics among NCAs at distinct temperatures. Two experimental batches with initial polymerization temperatures (IPT) below RT (viz., 14 and 18 °C) were produced. IPTs were maintained during 30 minutes, followed by a 30 min temperature increase until RT was reached and fixed for the rest of the polymerization, without further modifications over STD.

**Figure 3:**
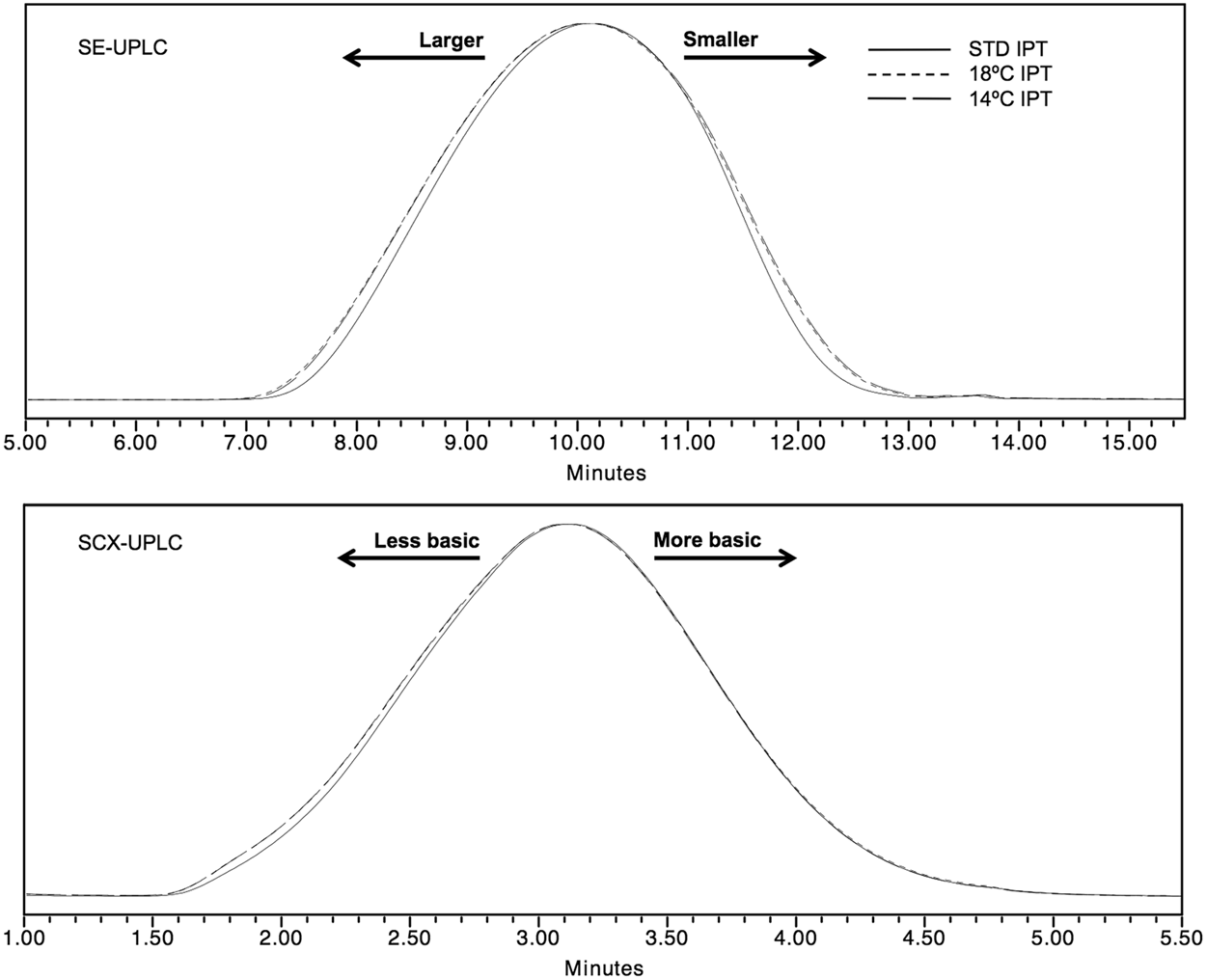
STD, 18 °C and 14 °C IPT experiments analyzed by SE-UPLC and SCX-UPLC.

It was revealed that IPTs increases GA distribution broadening, either for molecular mass or electric charge distributions (Fig. 3). This was in agreement with a previous report on polydispersity modulation at low temperature, in terms of end-group termination and side-reactions, which was hampered on low solubility copolymers^63^. In this sense, the low solubility of growing polymer chains at low temperatures causes a differential elongation behavior among them, thus leading to broader molecular mass and charge heterogeneities.

### Depolymerization time is related to abundance of GA smaller and less basic peptides

Following polymerization, cleavage of the synthesized polypeptides into smaller species was performed as part of GA manufacturing process. As expected, depolymerization conditions govern the extent at which GA polymers are cleaved^5,7,24–34^, which determine its physicochemical properties once the manufacturing process is finished.

**Figure 4:**
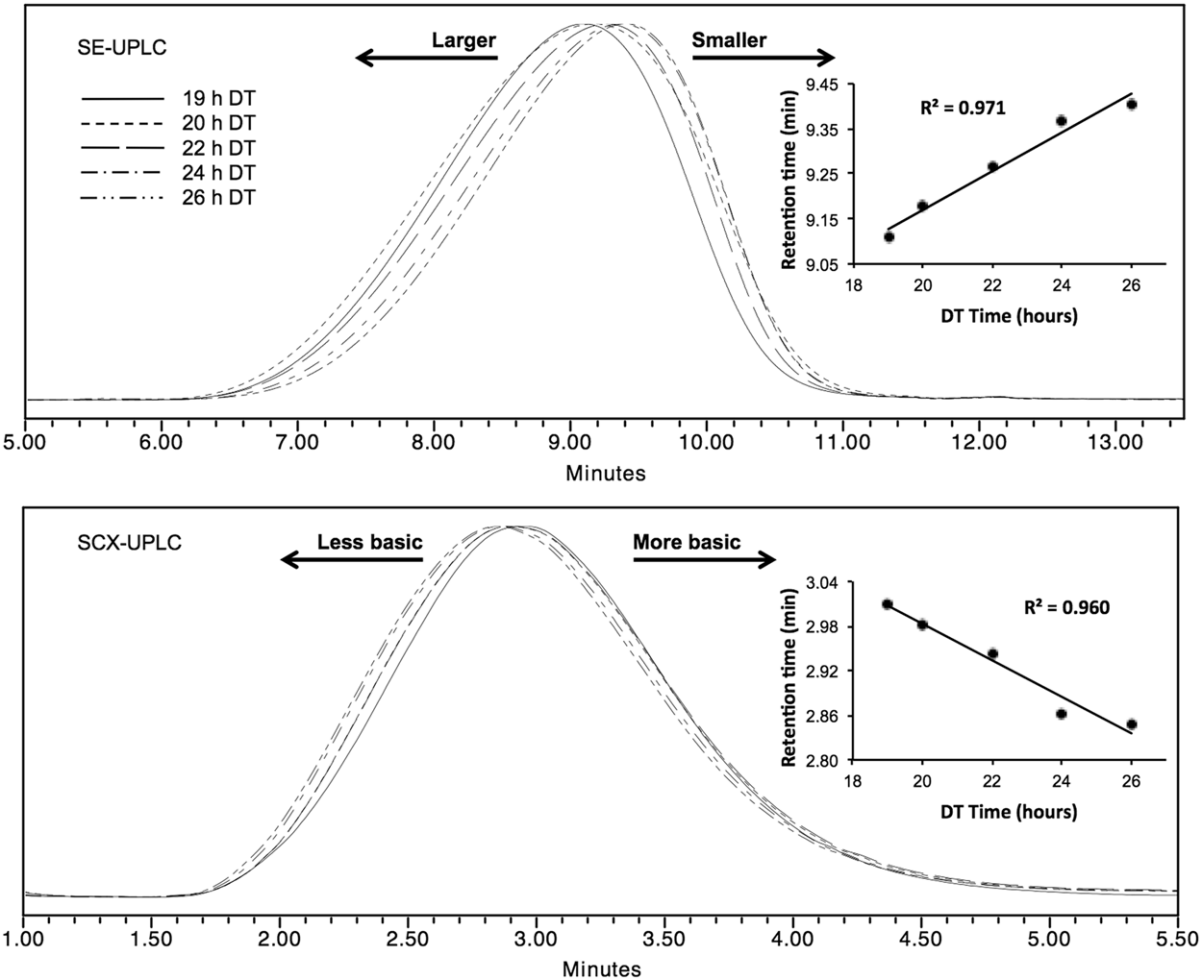
STD, 19 h, 20 h, 22 h, 24 h and 26 h DT experiments analyzed by SE-UPLC and SCX-UPLC. The insert shows the directly proportional correlation between retention time and DT by SE-UPLC, and its inversely proportional correlation by SCX-UPLC.

Naturally, depolymerization time (DT) was inversely proportional to the average molecular mass and electric charge of GA as seen by SE-UPLC and SCX-UPLC (Fig. 4). This relationship was assessed trough the comparison of the apex retention time in the chromatographic profiles and the DT used for each experimental batch. In both cases a linear relationship was observed with coefficient of determination (R^2^) values of 0.971 and 0.960 for SE-UPLC and SCX-UPLC, respectively (Fig. 4).

It is worth to notice that seminal process patents of GA lie on the assumption of this correlation, since an evaluation of DT is needed for every batch depending on the molecular weight obtained during polymerization^5,7,24–34^. However, as shown here, a well-defined polymerization process allows obtaining specific heterogeneity profiles, necessary for the establishment of fixed depolymerization conditions and to assure consistency on GA manufacturing process and identity.

### Membrane weight cut-off is related to abundance of GA smaller and less basic peptides

After depolymerization and deprotection steps, GA copolymer undergoes a membrane-based filtration process. This process has two functions, first, it serves as a washing step, by removing most of the process related impurities generated during the whole manufacturing process; and second, a purification process to achieve the desired GA heterogeneity by removing peptides in the low molecular mass range. Ultimately, resulting GA physicochemical properties are affected by the membrane weight cut-off (MWCO).

**Figure 5:**
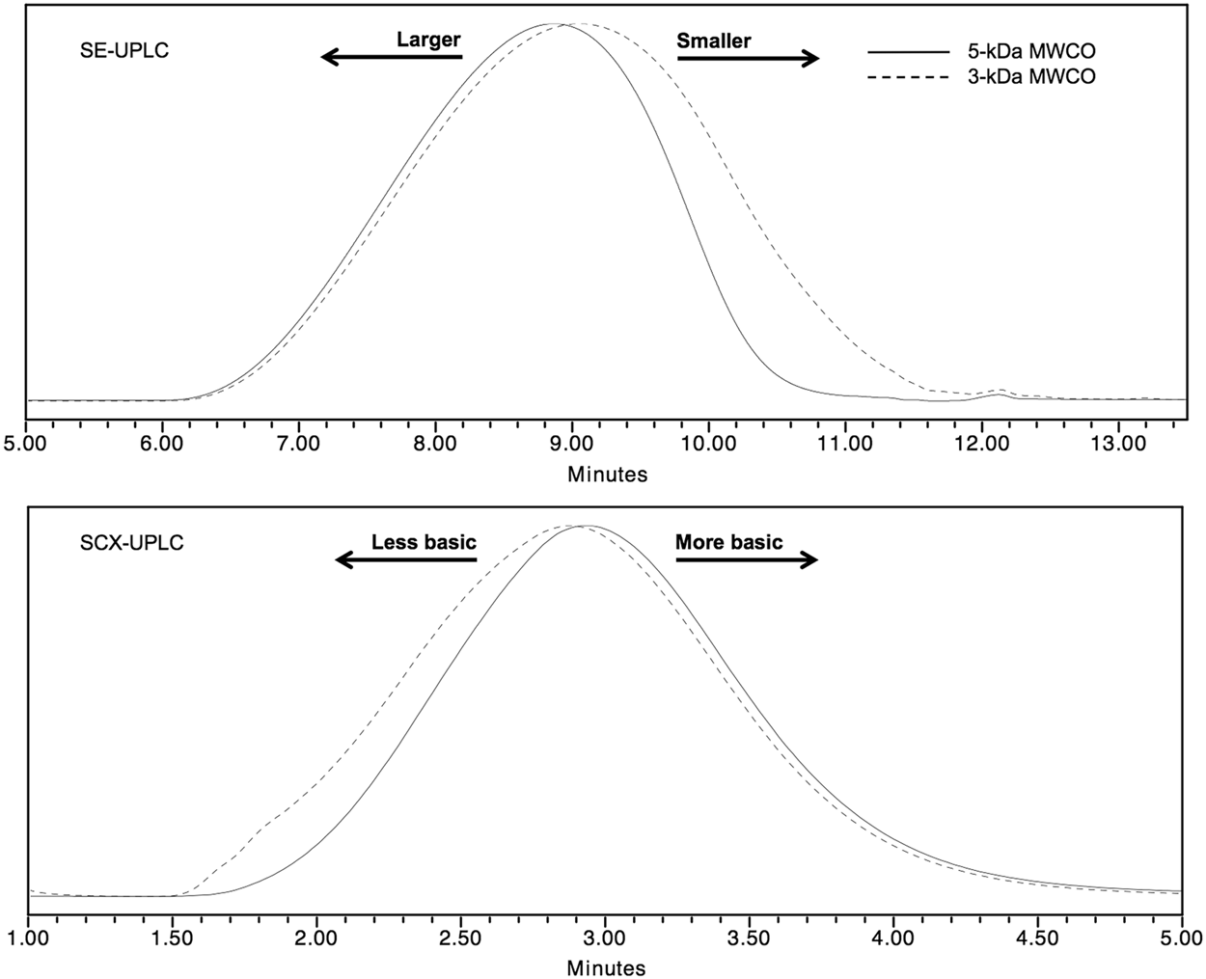
5-kDa and 3-kDa MWCO experiments analyzed by SE-UPLC and SCX-UPLC.

As expected, a 3-kDa MWCO membrane allowed to retain smaller and less basic peptides in comparison to a 5-kDa MWCO membrane (Fig. 5). On the other hand, the content of the larger and more basic peptides was only slightly altered between the two MWCO (Fig. 5). Confirming the selective removal of certain populations according to its size during this operation.

Overall, it has been shown how GA identity is governed by each stage of its manufacturing process. Critical process parameters must be understood and defined to assure obtaining the distinctive GA copolymer mixture, for instance: NCAs to DEA ratio, NCAs concentration and order of addition, polymerization and depolymerization time and temperature, as well as the MWCO used during diafiltration. The analyses discussed herein revealed that the heterogeneity profiles of the copolymers obtained during the distinct manufacturing conditions share sub populations of polypeptides with the same molecular masses or electric charges, but they can be distinguished by appropriate methodologies in terms of their differential global abundances. It is precisely the combination of those sub populations characteristics and their abundance that defines the structural signatures of GA and its identity, since deviations from the distinctive abundance of active epitopes responsible of its immunological mechanisms can significantly alter its safety and efficacy profiles^20,21^.

### GA immunogenic response is related to its whole heterogeneity

As mentioned, GA has an intricate nature as it is comprised of undefined chemical species that are degraded upon subcutaneous injection before exerting its therapeutic activities. This complexity links GA physicochemical properties with its pharmacological activity, thus indicating that non-GA heterogeneity profiles would have different therapeutic effects. To confirm the impact of modified GA heterogeneity profiles on the expected immunological behavior, GA-STD (Probioglat) and process-modified experimental batches were evaluated on its immunogenic response in comparison to the reference medicinal product (Copaxone), using a non-canonical immunization scheme in order to improve antigenic recognition by antigen-presenting cells, T- and B-cell cooperation, and the inflammatory environment. As intraperitoneal, subcutaneous and intramuscular administrations would stimulate intraperitoneal macrophages, promote the exposure to dendritic cells and favor the diffusion into nearby lymphatic nodes for recognition by B-cells, respectively^64^.

**Figure 6:**
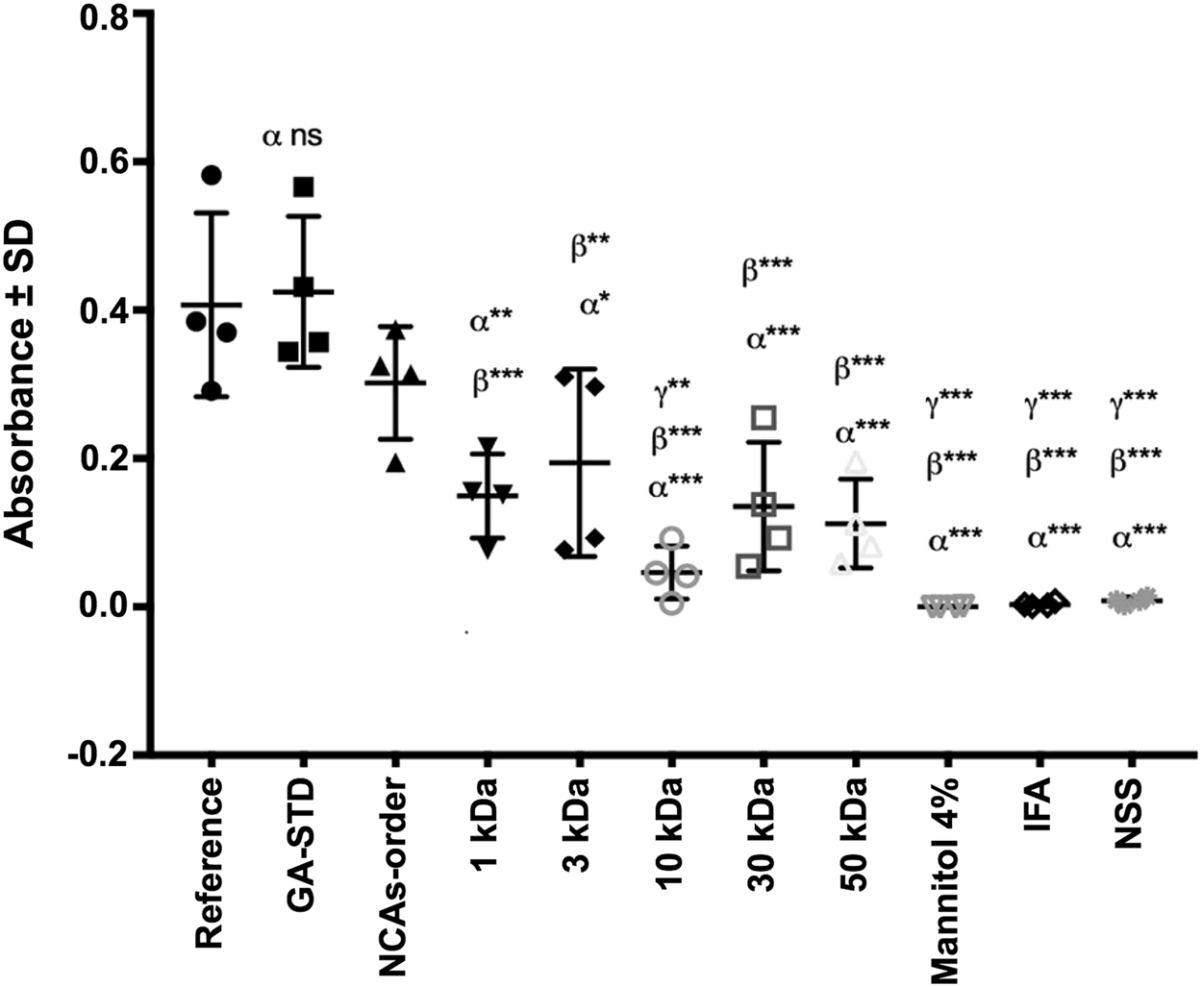
GA-specific antibodies detection of GA samples through ELISA. The statistical analysis included the comparison GA-specific antibodies production of each experimental group against the Reference Medicinal Product (α), the GA-STD (β) and NCA-order batch (γ) groups. *P < 0.05, **P < 0.001, ***P < 0.0001.

The results showed that after immunization with GA-STD or reference medicinal product batches, mice responded by producing anti-GA antibodies with an equivalent selectivity and affinity. This was observed as an analogous recognition of the mice sera obtained from GA-STD and reference medicinal product treatment groups against the reference medicinal product (i.e., an equivalent level of anti-GA antibodies). No significant difference between groups was determined by one-way ANOVA with Bonferroni *post hoc* test (Fig. 6).

Conversely, a lower recognition against the reference medicinal product from the mice sera obtained from process-modified experimental batches was observed, in comparison to mice sera obtained from GA-STD or reference medicinal product treatment groups (i.e., a non-equivalent level of anti-GA antibodies) (Fig. 6). It is worth to mention that an equivalent IgG concentration was obtained for all samples after the immunization period, ranging from 1 to 2 mg/mL, confirming that the observed immunogenic response differences were due to a distinct selectivity and affinity of the generated antibodies.

These results revealed that GA possesses a unique repertoire of active epitopes or amino acid sequences within its distinctive polypeptide mixture responsible of its immunological responses. This confirms that GA-STD and the reference medicinal product own the distinctive structural signatures that define GA identity, unlike process-modified experimental batches. In the latter case, with modifications on their amino acid sequence as revealed by their different molecular mass and electric charge heterogeneities that significantly alter its immunological profile by exposing a new or reduced (molecular mass fractionated batches) repertoire of epitopes to the immune system.

Besides the induction of antibodies in treated animals and patients^9–12^; it is known that GA biological activity is based on the ability to bias anti-inflammatory responses by inducing GA-specific T-cell activation, including a TH1 to TH2 phenotype shift^65–67^. Modifications on GA epitopes could lead to an altered response of T-cell receptors, which are highly sensitive to changes in the structure of the presented peptide ligands^68^, affecting activated T-cell repertoire and the global immunological profile^69^. Noticeably, if a manufacturing process is controlled in order to obtain a distinctive GA heterogeneity, the absence of modifications over the distinctive epitopes that populate GA and its biological outcomes must be expected.

For instance, these results agree with the differential immunogenic response observed for TV-5010^20,21^. Furthermore, these results revealed that GA immunogenic response depends on its whole polypeptide distribution instead of relying on a particular sub population of peptides.

### GA distinctive heterogeneity has the expected toxicology profile

In addition to the assessment of the immunological behavior of GA-STD and the reference medicinal product, single (acute) and multiple dose toxicity studies were performed in order to corroborate that a distinctive GA heterogeneity would lead to the expected toxicology profile. This study is relevant to assess the relationships between immunological responses, induction of toxic effects in the organism and the efficacy and safety results of a GA product from a specific manufacturing process.

Single subcutaneous administration toxicity studies in mice and rats revealed that a dose up to 400 mg/kg of GA-STD and the reference medicinal product was well tolerated, without mortality or clinical sings of toxicity. Similarly, no clinical signs of toxicity were observed for intravenous administration at a dose up to 300 mg/kg in mice and rats, with the exception of incidental effects at 200 and 300 mg/kg where transient prostration and dyspnea were observed on a male rat per group under GA-STD administration; without altering the conclusions of the study. Besides, in agreement with the toxicological information of GA at doses equal or higher to 400 mg/kg clinical signs and mortality were observed^58^, including hematomas in the inoculation zone and transient effects (prostration, dyspnea, exophthalmia, cyanosis in the tail and tremor) through the first minutes after administration were registered. At 400 mg/kg, mice mortalities of 20 and 30% were observed for GA-STD and the reference medicinal product, respectively; whereas at 500 mg/kg of both products a mortality of 100% was observed in mice and rats.

Regarding the multiple dose toxicity study at subcutaneous administration, it was revealed that a dose of 8 mg/kg/day, which is equivalent to a 4-fold increase of the recommended dose on humans^70^, was well tolerated during the 90 days of study. No mortality and no relevant clinical signs related to the treatments were detected either for GA-STD and the reference medicinal product. Weight gain was normal and no differences between the control (vehicle) and GA treatments groups were observed (p = 0.270, Greenhouse-Geisser test, software IBM SPSS 19). Accordingly, clinical biochemistry, hematology and urinalysis results indicated no relevant effects in the function of organs and systems (Table S1), as confirmed by necropsy and histopathology (data not shown).

Overall, acute and multiple dose toxicology profiles results were within the expected forecast. These results, in combination with reported toxicological effects of TV-5010^20,21^, confirmed that distinctive structural signatures of GA outline its safety profile.

In conclusion, this systematic research describes a novel approach to determine how critical process parameters impact on GA distinctive heterogeneities and its identity. Moreover, it gives insights on relevant GA structural and functional relationships. In effect, our findings revealed that assessing the process signatures of GA by its heterogeneity profiles is useful to determine a particular GA immunogenic response, within the several proposed immunological mechanisms, utterly responsible of its therapeutic activity. Further, distinctive structural signatures of GA outline its safety profile as seen by their expected toxicological profiles. This provides advance knowledge on GA identity towards enriching its comprehensiveness and ensuring consistent manufacturing of safe and effective polypeptide MS treatments.

## Materials and Methods

### Chemicals and Reagents

All chemicals and reagents used for samples preparation and analyses were ACS grade or greater from J.T. Baker (Avantor Performance Materials, Inc., Center Valley, PA) or Sigma Aldrich (St. Louis, MO). All assays were performed using ultrapure Milli-Q water (Millipore, Illkirch-Graffenstaden, France).

### Glatiramer Acetate samples

Glatiramer acetate produced under standard synthesis conditions (GA-STD, Probioglat) drug substance and drug product (GA 20 mg/mL solution for subcutaneous use) were obtained from Probiomed S.A. de C.V. (Mexico City, Mexico) while Copaxone (GA 20 mg/mL solution for subcutaneous use) was obtained from Teva Pharmaceutical Industries (Central District, Israel).

#### Process-modified samples

Standard synthesis conditions (STD) for GA, used for the study of critical process parameters, were established by following the basic framework of the well-known procedure initially reported by Teitelbaum *et al*.^7^.

Further, critical process parameters were evaluated by systematically modifying the STD on a set of tailored experimental batches. These batches were synthesized with a single and particular modification, leaving remaining conditions unchanged. The particular modifications used for the process-modified samples are described in the results and discussion segment.

A tailored GA batch synthesized by changing the NCAs order (called as NCAs-order batch) was used for the immunological study. The NCAs-order batch was synthesized by initiating the polymerization with NCA-glutamic acid and one third of the NCA-lysine and NCA-alanine material only, and adding the remaining material along with NCA-tyrosine after 7 minutes, followed with the standard process.

#### Molecular mass fractionated samples

Molecular mass fractionated samples of GA were generated by serial fractionation using modified polyethersulfone cassettes with 50 kDa, 30 kDa, 10 kDa, 3 kDa and 1 kDa nominal molecular weight cut-offs (Omega Centramate 0.1 SQ M/1.1 SQ.FT) from Pall Corporation (New York, NY). 10 g of lyophilized GA were reconstituted in water to 65 mg/mL and then filtered with a 50 kDa cassette coupled to a Crossflow Filter Holder (Sartorius Stedim Biotech, Aubagne, France). The retained solution was washed with water until the permeate absorbance at 275 nm was lower than 0.05 UA. Collected permeate was used to obtain the subsequent fractions at 30 kDa, 10 kDa, 3 kDa and 1 kDa using the same procedure. The complete fractionation process was performed in a class II biological safety cabinet (The Baker Company, Sanford, ME) and the endotoxin levels were kept below 0.25 endotoxin units per milliliter as checked before and after each step by a gel clot method.

### Size Exclusion Ultra Performance Liquid Chromatography (SE-UPLC)

Samples treatment and analysis conditions for SE-UPLC were performed as previously described by Espinosa-de la Garza *et al*.^45^.

### Strong Cation Exchange Ultra Performance Liquid Chromatography (SCX-UPLC)

Samples treatment and analysis conditions for SCX-UPLC were performed as previously described by Campos-García *et al*.^46^.

### Immunological assay

#### Mice immunization

The immunological study was performed in male Balb/C mice, aged 6–8 weeks and 15–20 g weight (Ferandelh, Mexico City, Mexico), in accordance with the Mexican guidelines on the use and care of laboratory animals^71^ and the International Guide for the Care and Use of Laboratory Animals^72^. Mice were housed in a MicroVent system (Allentown Inc, Allentown, NJ) with food (Envigo, Cambridgeshire, United Kingdom) and water *ad libitum*. All procedures were approved by the CIPFT research committee under code FTU/DF 015/001/PRO and all efforts were made to minimize animal suffering and reduce the number of animals per assay.

Mice were divided into eight experimental and three control groups, including six and three mice per group, respectively. Each tested antigen was prepared as a mixture with Incomplete Freund Adjuvant (IFA) at 1:1 ratio to a final concentration of 5 µg/µL. 40 µL of the tested antigen mixture were administrated for each experimental group. Experimental groups were treated independently with: GA obtained by STD manufacturing process (Probioglat, batch: 4363151011); reference medicinal product (Copaxone, batch: p63168); NCAs-order batch; and 1, 3, 10, 30, 50 kDa-fractionated samples. Control groups were immunized independently with 40 µL of GA drug product excipient (4% w/v solution of mannitol), IFA and normal saline solution (NSS).

Scheme of immunization consist of intraperitoneal, subcutaneous and intramuscular administrations on day 0, 7 and 14, respectively. Blood samples were collected at day 21 from the facial vein in Microtainer tubes (BD Biosciences, Franklin Lakes, NJ). Mice serum were obtained by centrifugation at 670 × g for 15 min and stored at −80 °C until analysis. None of the experimental animals exhibited adverse effects or died during the study.

#### Total IgG quantitation

Mice sera of each immunization group were thawed at room temperature and pooled. Then, 20 µL were diluted with 40 µL of a 20 mM phosphate, 200 mM NaCl buffer solution at pH 7.0, and filtered through a 0.10 µm PVDF membrane (Merck Millipore, Hess, Germany). Samples were analyzed by triplicate on a Poros A/20 affinity column (2.1 mm × 30 mm) (Applied Biosystems, Foster City, CA) coupled to an Acquity UPLC system (Waters, Milford, MA). Retained IgGs were eluted by a linear gradient from 20 mM phosphate, 200 mM NaCl buffer solution at pH 7.0 to a 20 mM phosphate buffer solution at pH 3.0 with a flow rate of 0.5 mL/min. Detection was performed on an Acquity UPLC UV Detector at 280 nm (Waters, Milford, MA). Column and sample temperatures were maintained at 28 and 10 °C, respectively. IgGs were quantitated by standard curve using a murine IgG standard (BD Biosciences, Franklin Lakes, NJ).

#### GA-specific antibodies detection and quantitation

Specific antibodies against GA were detected and quantitated by an enzyme-linked immunosorbent assay (ELISA). ELISA plates were sensitized with reference medicinal product at 20 µg/mL by overnight incubation at 4 °C. This was followed by incubation of mice sera from each immunization group at 1:2000 dilutions. Detection of GA-specific antibodies was achieved using a primary anti-mouse IgG, and a secondary (H + L)+ Biotin goat anti-mouse IgM (Thermo Scientific, Waltham, MA) at 1:10000 and 1:1000 dilutions, respectively. Assay revealing was performed with streptavidin-HRP (BD Biosciences, Franklin Lakes, NJ) using an EPOCH spectrophotometer (Biotek Instruments, Winooski, VT) at 450 nm/570 nm. Results were processed using GraphPad Prism software v.5.00 (GraphPad Software, San Diego, CA). Statistical one-way ANOVA with Bonferroni *post hoc* tests were conducted at a 0.05 level of significance.

### Acute and multiple dose toxicity profile of GA

Subcutaneous and intravenous single dose toxicities were evaluated in mice (Hsd:ICR) and in Wistar rats (Hsd:WI). Multiple dose toxicity was assessed at subcutaneous route in Wistar rats (Hsd:WI). All the procedures were performed in compliance with the Mexican guidelines on the use and care of laboratory animals^71^ and the Instituted Committee of Animal Care and Use of the Faculty of Chemistry of the National Autonomous University of Mexico (UNAM) approved the protocols. The animals were bred by ENVIGO barrier 650 (Mexico City, Mexico), and housed under a controlled environment (temperature of 22 ± 2 C°, relative humidity of 40–60% and 12 hours light/dark cycles) with Teklad 2018S food (Envigo, Cambridgeshire, United Kingdom) and water *ad libitum*. All efforts were made to minimize animal suffering and reduce the number of animals per assay. Experimental groups were treated with GA-STD (Probioglat, batch: 4363P0903E) and reference medicinal product (Copaxone, batch: P53449).

The dose levels and conditions used for the single dose (acute) and multiple dose toxicity studies were performed according with previous studies developed with the reference medicinal product^40^. Mice and rats groups (10 animals per group, 5 females and 5 males per group) included in the subcutaneous single dose toxicity study were administered with 400 mg/kg of GA sample in the dorsal zone (representing 100 and 200 fold the recommended human dose, based on mg/m^2^ in mouse and rat respectively). Intravenous route study in mice and rats (10 animals per group, 5 females and 5 males per group) included doses of 100, 200, 300, 400 and 500 mg/kg of GA samples through the caudal vein. All the animals were monitored through 14 days after the administration, including the recording of clinical signs and body weight.

Multiple dose toxicity study was assessed in male Wistar rats (13 animal per group) trough subcutaneous administration (dorsal zone) of 8 mg/kg/day of GA samples based on the reported NOAEL of the reference medicinal product^69^. Study last 90 days, including periodical recording of clinical signs and body weight. Standard parameters of clinical biochemistry, hematology and urinalysis were evaluated prior first administration and on the days 30, 60 and 90. At the end of the study the animals were euthanized with carbon dioxide and necropsied, including histopathological analyses.

### Data availability

All data generated or analyzed during this study are included in this published article (and its Supplementary Information files).

## Electronic supplementary material

Table S1
