# Supplementary material for: Process signatures in glatiramer acetate synthesis: structural and functional relationships
Source: Sci Rep. 2017 Sep 21;7:12125. doi: 10.1038/s41598-017-12416-1 (PMC5608765; doi:10.1038/s41598-017-12416-1)
Supplement: Supplementary file 1 — Table S1 [file 41598_2017_12416_MOESM1_ESM.pdf]

Process signatures in glatiramer acetate synthesis: structural and functional relationships

Víctor R. Campos-García[1], Daniel Herrera-Fernández[1], Carlos E. Espinosa-de la Garza[1], German González[1], Luis Vallejo-Castillo[2,3], Sandra Avila[2], Leslie Muñoz-García[2], E. Medina-Rivero[2], Néstor O. Pérez[1], Isabel Gracia-Mora[4], Sonia Mayra Pérez-Tapia[2,5], Rodolfo Salazar-Ceballos[1], Lenin Pavón[6], Luis F. Flores-Ortiz[1]*

[1] Unidad de Investigación y Desarrollo, Probiomed S.A. de C.V., Cruce de Carreteras Acatzingo-Zumpahuacán s/n, Colonia Los Shiperes, 52400, Tenancingo, Estado de México, México.

[2] Unidad de Desarrollo e Investigación en Bioprocesos (UDIBI), Escuela Nacional de Ciencias Biológicas, Instituto Politécnico Nacional, Prolongación de Carpio y Plan de Ayala s/n, Colonia Santo Tomás, 11340, Ciudad de México, México.

[3] Departamento de Farmacología, Cinvestav-IPN, Avenida Instituto Politécnico Nacional 2508, Colonia San Pedro Zacatenco, 07360, Ciudad de México, México.

[4] Departamento de Química Inorgánica y Nuclear, Facultad de Química, Universidad Nacional Autónoma de México (UNAM), Ciudad Universitaria, Investigación Científica 70, 04510, Ciudad de México, México.

[5] Unidad de Investigación, Desarrollo e Innovación Médica y Biotecnológica (UDIMEB), Escuela Nacional de Ciencias Biológicas, Instituto Politécnico Nacional, Prolongación de Carpio y Plan de Ayala s/n, Colonia Santo Tomás, 11340, Ciudad de México, México.

[6] Laboratorio de Psicoinmunología, Dirección de Investigaciones en Neurociencias, Instituto Nacional de Psiquiatría Ramón de la Fuente, Calzada México-Xochimilco 101, Colonia San Lorenzo Huipulco, 14370, Ciudad de México, México.

***Corresponding author**

Luis F. Flores-Ortiz

Postal address: Cruce de Carreteras Acatzingo-Zumpahuacán s/n, 52400, Tenancingo, Estado de México, México.

e-mail: luis.flores@probiomed.com.mx

Fax: +52 (55) 5352-7651

Tables

Table S1. Clinical biochemistry, hematology and urinalysis parameters at day 90 of the multiple dose toxicity study. Data are presented as mean ± standard error from 13 animals/group.

**Parameter**	**Range of normal values**	**Control**	**GA-STD**	**Reference medicinal product**
**Clinical biochemistry**				
Albumin	40.3 – 49.3 g/L	39.51 ± 0.22	37.83 ± 0.22	40.48 ± 0.22
Urea	2.47 – 11.30 mmol/L	6.48 ± 0.10	6.06 ± 0.10	6.24 ± 0.10
Alkaline phosphatase	170 -309 U/L	394.43 ± 18.8	390.58 ± 18.8	406.80 ± 18.8
Alanine aminotransferase	34 – 83 U/L	51.96 ± 1.25	47.76 ± 1.25	54.63 ± 1.25
Aspartate aminotransferase	88 -215 U/L	129.00 ± 5.08	150.91 ± 5.08	166.77 ± 5.08
Total bilirubin	1.47 – 7.18 μmol/L	4.18 ± 0.10	3.77 ± 0.13	3.79 ± 0.13
**Hematology**				
Hematocrit	0.45 – 0.52/L	0.49 ± 0.004	0.50 ± 0.004	0.50 ± 0.004
Hemoglobin	137 – 200 g/L	167.07 ± 1.16	165.78 ± 1.16	167.88 ± 1.16
Erythrocytes	6.5 – 9.6 x 10^{12}/L	8.62 ± 0.06 x 10^{12}	8.63 ± 0.06 x 10^{12}	8.74 ± 0.06 x 10^{12}
Platelet counts	474 – 1177 x 10^{9}/L	464.20 ± 15.4 x 10^{9}	468.8 ± 15.4 x 10^{9}	480.2 ± 15.4 x 10^{9}
Leukocyte	6.6 – 20.3 x 10^{9}/L	8.89 ± 0.32 x 10^{9}	9.14 ± 0.32 x 10^{9}	8.84 ± 0.32 x 10^{9}
Neutrophils	0.4 – 2.6 x 10^{9}/L	1.49 ± 0.14 x 10^{9}	1.53 ± 0.14 x 10^{9}	1.60 ± 0.14 x 10^{9}
Lymphocytes	6.1 – 1.8 x 10^{9}/L	7.20 ± 0.33 x 10^{9}	7.40 ± 0.33 x 10^{9}	7.02 ± 0.33 x 10^{9}
Monocytes	0.0 – 0.05 x 10^{9}/L	0.032 ± 0.02 x 10^{9}	0.11 ± 0.02 x 10^{9}	0.09 ± 0.02 x 10^{9}
Eosinophils	0.0 – 0.3 x 10^{9}/L	0. 26 ± 0.01 x 10^{9}	0.029 ± 0.01 x 10^{9}	0.031 ± 0.01 x 10^{9}
**Urinalysis**				
Protein	Negative or trace	Negative	Negative	Negative
Glucose	Negative or trace	Negative	Negative	Negative
Bilirubin	Negative	Negative	Negative	Negative
